# Dental follicles promote soft tissue management in surgical exposure of labially impacted maxillary canine

**DOI:** 10.1186/s12903-021-01922-4

**Published:** 2021-11-08

**Authors:** Li-Ru Hu, Wen-Ting Qi, Chong-Yun Bao, Jian Pan, Xian Liu

**Affiliations:** State Key Laboratory of Oral Diseases, and National Clinical Research Center for Oral Diseases, and Department of Oral and Maxillofacial Surgery, West China Hospital of Stomatology, Sichuan University, #14 Third Section, Renmin Road South, 610041 Chengdu, China

**Keywords:** Surgical exposure, Impacted tooth, Orthodontic, Periodontal status

## Abstract

**Background:**

The present study aimed to report a technically improved operation on the surgical exposure of labially impacted maxillary canine, elaborating the management of soft tissue to achieve better aesthetic results, and post-treatment periodontal health.

**Methods:**

Patients sought orthodontic treatment with unilateral labially impacted maxillary canines were selected in this study. The impacted teeth were assigned to the experimental group and contralateral unimpacted canines were assigned to the control group. The impacted canines were surgically exposed with dissected dental follicle (DF) stitching to muscle and mucosa surrounding the crowns. The gingival index (GI), probing depth (PD), the width of the keratinized gingiva (WKG), gingival scars (GS), bone loss (BL), and apical root resorption (ARR) were recorded after the removal of the fixed appliance. A two-sample t-test was used for independent samples for parametric variables.

**Results:**

A total of 24 patients with unilateral maxillary canine impaction were successfully treated. The outcomes of GI, WKG, GS, BL, and ARR did not indicate statistical significance between the experimental group and the control group.

**Conclusions:**

The preservation of DF promotes soft tissue management in combined surgical and orthodontic treatment of labially impacted maxillary canine to achieve better periodontal status.

*Trial Registration* Chinese Clinical Trial Registry ChiCTR2000029091, 2020-01-12.

## Background

The maxillary canines are crucial for maxillofacial aesthetics and dental occlusion. Their impaction, although rare, can severely affect oral function, and an occlusal relationship that reduces the quality of life [1, 2]. A combined treatment of surgical exposure and orthodontic therapy is a viable alternative to early interceptive measures with extraction of deciduous teeth failing to prevent the development of impacted anterior teeth [3, 4].

Surgical exposure treatments include two major approaches: the closed-eruption technique (CET), and the open-eruption technique (OET) [5]. Surgical flaps are repositioned leaving the crown invisible after soft tissues and bone tissue covering the crown are removed in CET [6]. If the orthodontic attachment falls off, secondary exposure surgery is required [7]. In contrast, using the OET the crown remains clinically visible by the use of an apically repositioned gingival flap, or removal of covered mucoperiosteum to let the impacted tooth erupt spontaneously; or an orthodontic attachment can be directly bonded to the impacted tooth to apply traction directly. One of the disadvantages is an open wound might cause severe damage and discomfort [8]. In some cases of spontaneous eruption, the risk of mucosal re-covering and insufficient removal of bone might necessitate retreatment.

Whether using the OET or the CET, the emphasis of surgical treatment is exposure of the crown to produce a feasible eruption pathway, and unfortunately tissue management can be neglected during surgery. Surgical damage and tissue removal can bring about poor periodontal status and unaesthetic sequalae, including gingival recession and scarring [9, 10]. Woloshyn et al. reported a significantly deeper probing depth (impacted teeth: 3.06 mm; normally erupted teeth: 2.69 mm), and crestal bone loss (impacted teeth: mesial 0.98 mm and distal 1.25 mm; normally erupted teeth: mesial 0.46 mm and distal 1.02 mm versus), of impacted canines that underwent surgically assisted orthodontic traction [11].

During tooth eruption, the dental follicle (DF) initiates the resorption of the roots of deciduous teeth and bone to create an eruption pathway [12]. The DF is a loose connective tissue layer surrounding the cementoenamel junction (CEJ) of the erupting tooth germ. Stem cells and precursor cells originating from the DF differentiate into osteoblasts, cementoblasts and periodontal ligament fibroblasts that form periodontium including the alveolar bone, cementum and periodontal ligament [13, 14]. A set of molecules such as the receptor activator of nuclear factor kB (RANK) and the colony-stimulating factor-1 (CSF-1) regulate bone remodeling [15]. The DF has been shown to be a source of RANK, and mesenchymal progenitor cells in the DF surrounding a tooth differentiate into various cells which compose the root-bone surface [16]. Previous studies have shown that the use of dental follicle cells as seed cells had superior biological properties and resulted in better periodontal tissue regeneration in vitro compared to other strategies [17, 18]. Therefore, the DF is considered to play an important role in tooth eruption and periodontium formation.

The aim of this manuscript is to report a technically improved operation on the surgical exposure of labially impacted maxillary canine.

## Methods

**Fig. 1:**
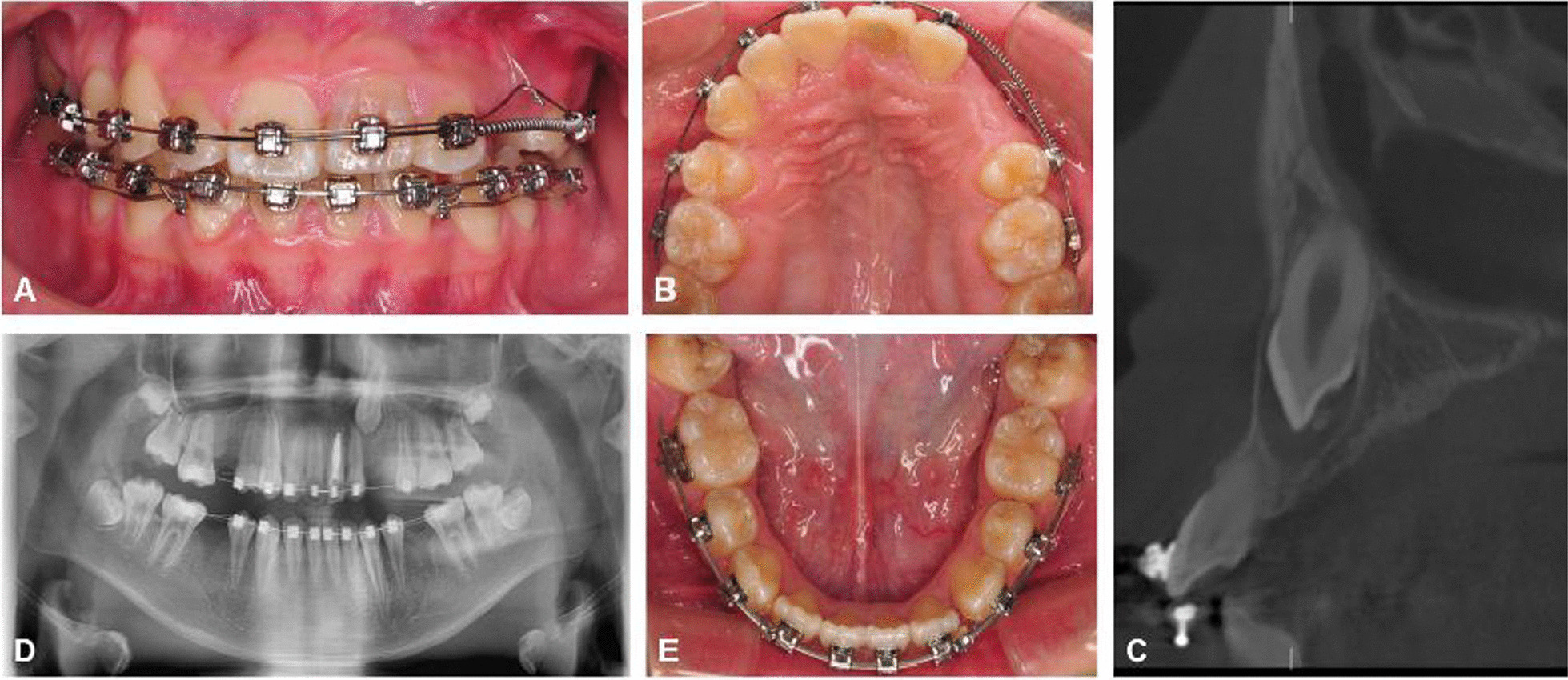
Evaluation of the impacted teeth: clinical photographs (**A**, **B**, **E**), CT scan (**C**) and orthopantomogram (**D**)

The present study was approved by the Ethics Committee of West China Hospital of stomatology (WCHSIRB-CT-2020-212). In this study, we identified 24 consecutive patients (15 females and 9 males) with a mean age of 14.5 (range from 10 to 29 years) who underwent surgical exposure treatment for unilateral labially impacted maxillary canine and orthodontic treatment in the West China Hospital of stomatology (Fig. 1). All patients were not accompanied by periodontal breakdown or history of periodontal disease. All Patients received sufficient explanations regarding the purpose, the benefits, and possible risks of this clinical research, and informed consent was obtained from each patient. Patient information including gender, and systemic conditions was collected. The impacted teeth were assigned to the experimental group and contralateral unimpacted canine teeth were assigned to the control groups. After the completion of fixed orthodontic treatment, two blinded dental investigators (Li-Ru Hu and Wen-Ting Qi) measured the periodontal status including gingival index (GI), probing depth (PD), the width of the keratinized gingiva (WKG) [19], gingival scars (GS) [9], bone loss (BL) [20], and apical root resorption (ARR) [21].

### Periodontal evaluation

GI was scored on the mesial, distal, buccal, and palatal surfaces; and mean gingival indices were determined for each tooth. The GI was scored as follows: 0 = normal gingiva, 1 = mild inflammation, slight change in color, slight edema, no bleeding on probing, 2 = moderate inflammation, redness, edema, glazing, bleeding on probing, and 3 = severe inflammation, marked redness and edema, ulcerations, tendency toward spontaneous bleeding [22]. PD was examined with a standard periodontal probe at the mesiolabial, midlabial, distolabial, mesiopalatal, midpalatal, and distopalatal surfaces for each tooth. A mean PD was calculated for each site. The distance from the free gingival margin to the mucogingival junction defined KG. Gingival scars were defined as noticeable soft tissue bands. The presence of any gingival scars around the teeth in the experimental group were recorded.

The cemento-enamel junction (CEJ) and alveolar crest were defined on the median section of the crown. Alveolar bone loss was calculated from the CEJ to the alveolar crest on the cone-beam computed tomography (CBCT). Root length was measured from the point of the cusp to the apex using the CBCT, and ARR on each tooth was defined as the difference of tooth length before and after orthodontic treatment.

### Surgical technique

**Fig. 2:**
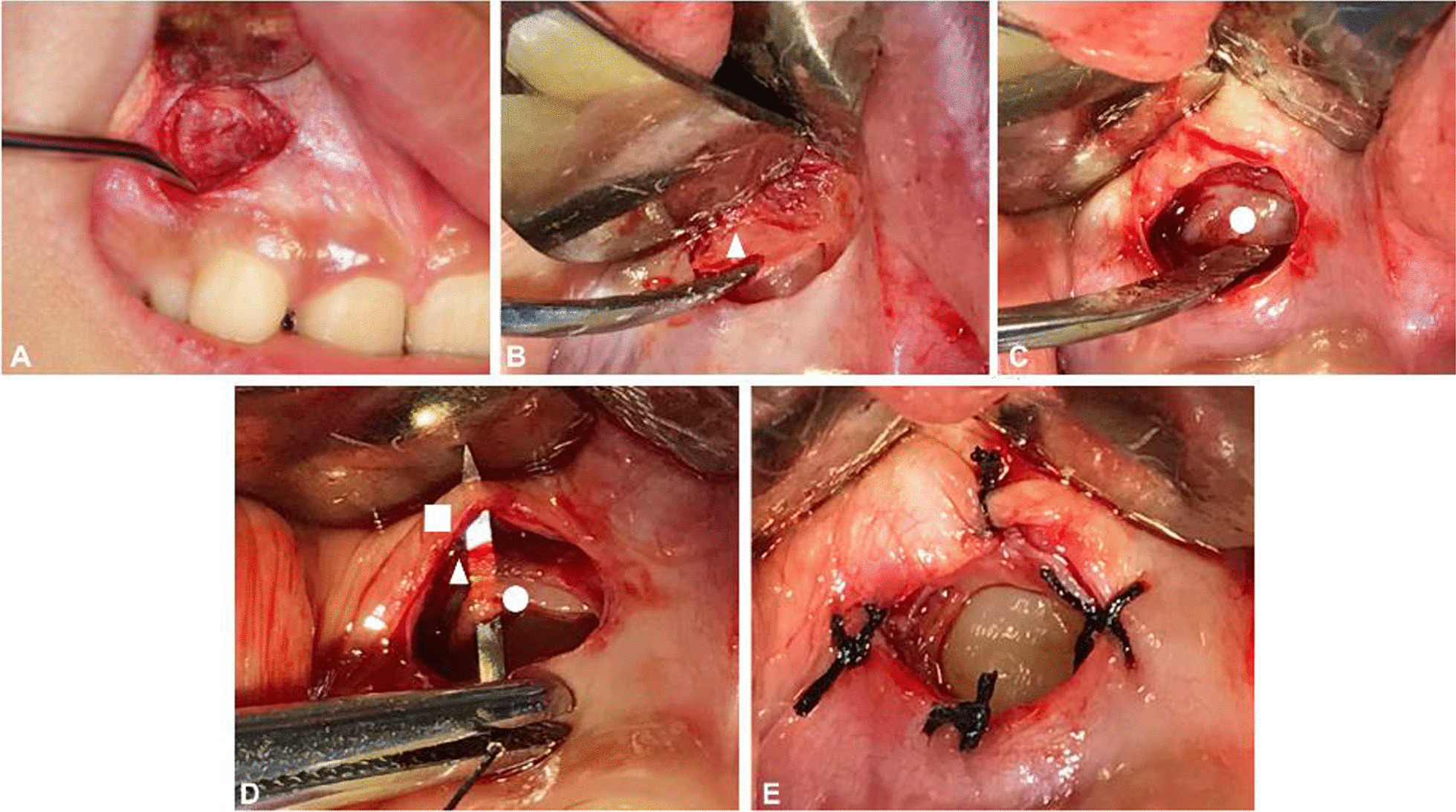
Incision and full thickness flap reflection (**A**). Bone removal: the triangle indicates the bone surface (**B**). Incision of the dental crown: the circle indicates the dental follicle (**C**). Suture: the circle indicates the dental follicle; the triangle indicates muscle; the square indicates the mucosa (**D**, **E**)

The exposure of the impacted tooth was performed under local anesthetic with a precise nerve block of nasopalatine and labial infiltration to obtain adequate anesthesia, and Fig. 2 presents a typical case.

In the experimental group, a curved incision was undertaken on the mucosa within the scale of the surface of the crown, and the location of incision was estimated roughly in CBCT. The submucosal tissues and muscle were incised step by step to expose the bone surface thoroughly (Fig. 2A). The bone covering the crown was carefully removed with a punch or bur, without damaging subjacent DF (Fig. 2B). The surgical field was washed with normal saline irrigation in order to provide a clear view of intact DF. The curved incision was performed on the incisal one-third of the labial surface of the DF parallel to the incisal ridge to obtain adequate exposure (Fig. 2C). Dissected DF and mucosa were sutured with a 5–0 nylon suture surrounding the crown (Fig. 2D, E). An antiseptic gauze roll was then put on the wound for compression hemostasis.

**Fig. 3:**
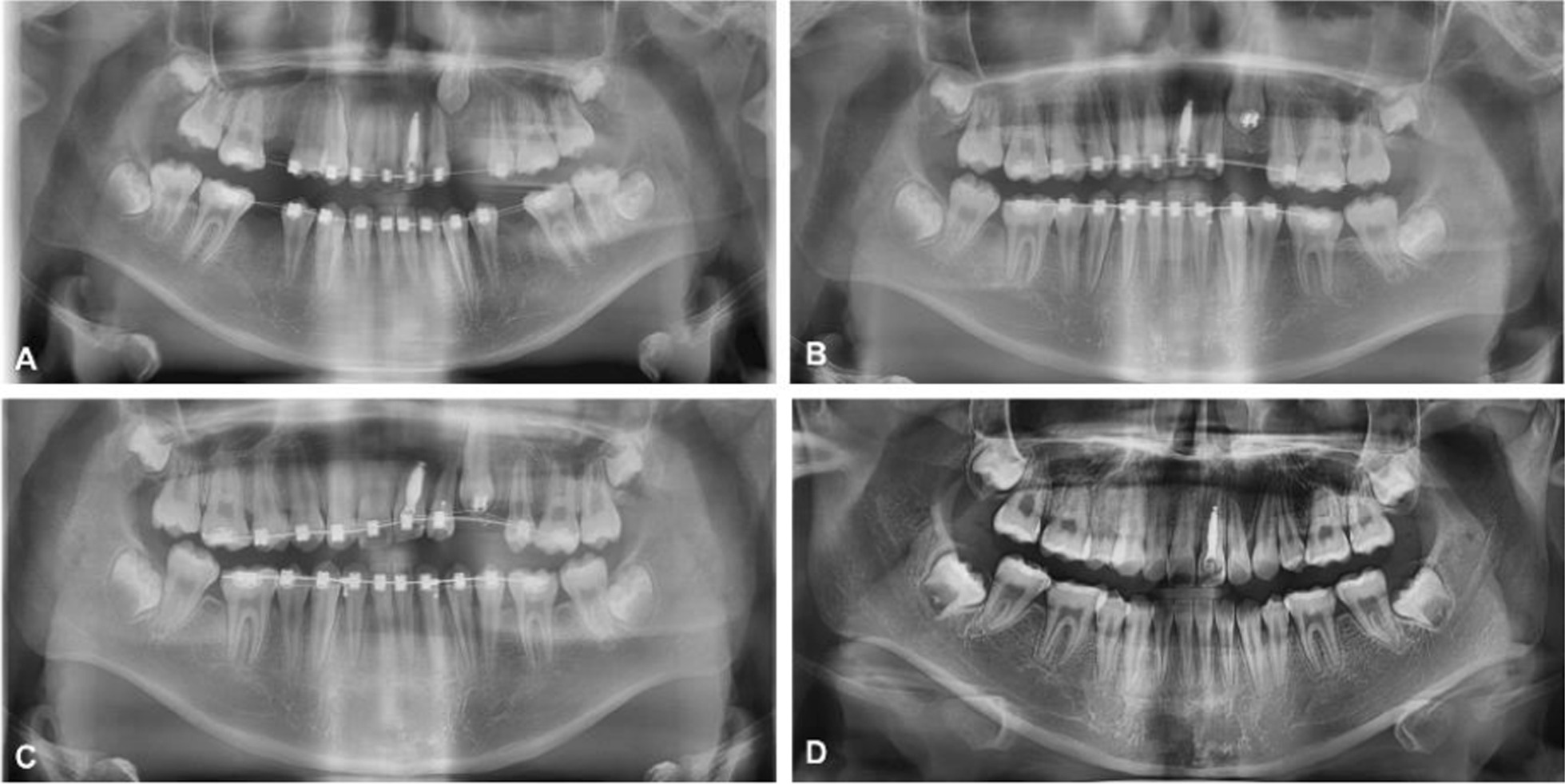
Orthopantomograms during orthodontic treatment (**A**–**D**)

**Fig. 4:**
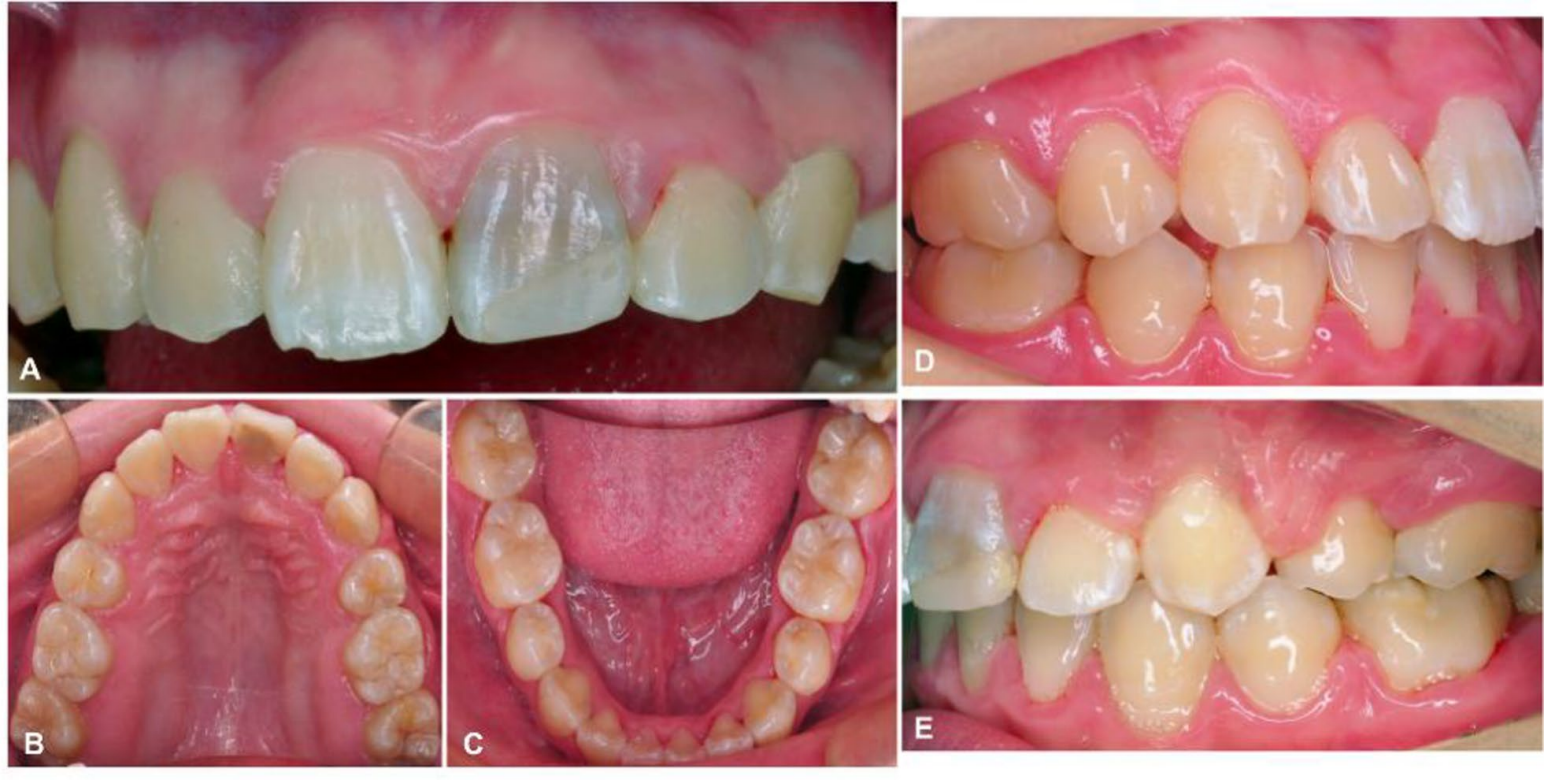
Clinical evaluation post-orthodontic treatment (**A**–**E**)

Preadjusted edgewise metallic brackets of 0.022 × 0.028-in. slot were used. For canine traction, a 0.018 × 0.025-in. rectangular nickel-titanium archwire was utilized to maintain the arch shape, while a 0.014-in. nickel-titanium archwire provided the traction force. Since the root of maxillary left lateral incisor and the crown of maxillary left canine might collide during orthodontic tooth movement, sequential traction was necessary: firstly, distal movement of the maxillary left canine was performed, moving its crown away from the root of the lateral incisor. Then, occlusal traction of the impacted canine was completed. After sequential traction, the impacted canine was brought to the arch to achieve preliminary alignment. When orthodontic traction was begun, the eruption status was precisely documented at every regular reexamination (Fig. 3). Periodontal and bony measurements were recorded for the impacted tooth and the contralateral tooth at the time of removal of fixed appliance (Fig. 4).

### Data analysis

Statistical analysis was performed using statistical software SPSS (version 20.0, IBM Corp., Armonk, NY, USA). A two-sample t-test was used for independent samples for parametric variables. All *P* values were two-tailed, and data statistical significance was set at *P* < 0.05.

## Results

A total of 24 patients with unilateral maxillary canine teeth impaction were successfully treated. Only 3 patients (12.5%) reported mild pain on the first postoperative day while no patient reported obvious swelling and inflammation. Gingival scars were observed in 2 patients in the experimental group. Generally, the scores for gingival index in the experimental group was 0.47 while in the control group was 0.45. Significant pathologic periodontal diseases were not observed by visual examination or periodontal probing (Table 1). The mean duration of treatment from surgical exposure to alignment of the impacted canines in the dental arch was 9 months ranging from 4 to 18 months.

**Table 1:** Probing depth and the width of keratinized gingiva in the two groups

Measurement	Impacted canines			Contralateral canines			*P* value
	Mean (mm)	SD	Range	Mean (mm)	SD	Range	
*PD*							
Mesiolabial	2.61	0.27	2.2–3.2	2.45	0.27	2.1–3.1	0.04*
Midlabial	1.60	0.23	1.3–2.1	1.48	0.21	1.2–2	0.083
Distolabial	2.70	0.27	2.2–3.4	2.53	0.25	2.3–3.2	0.03*
Mesiopalatal	2.61	0.26	2.2–3.2	2.49	0.22	2.1–3	0.079
Midpalatal	1.45	0.23	1.1–2	1.30	0.23	1–1.8	0.034*
Distopalatal	2.72	0.28	2.4–3.5	2.61	0.28	2.2–3.4	0.187
WKG	4.04	0.52	3.1–5.1	4.24	0.51	3.2–5.3	0.193

*p < 0.05

*PD* probing depth, *WKG* the width of the keratinized gingiva

In general, PD was higher in the experimental group than in the control groups. The difference was statistically significant at the mesiolabial site (experimental group: 2.61 ± 0.27 mm, control group: 2.45 ± 0.27 mm; *P* = 0.04), distolabial site (experimental group: 2.70 ± 0.27 mm, control group: 2.53 ± 0.25 mm; *P* = 0.03), and midpalatal site (experimental group: 1.45 ± 0.23 mm, control group: 1.30 ± 0.23 mm; *P* = 0.034). Mean KT was generally lower in the experimental group than in the control groups. This difference showed no statistical significance between experimental group and control group (*P* = 0.193) (Table 2).

**Table 2:** Treatment-related bone loss and apical root resorption

Measurement	Impacted canines			Contralateral canines			*P* value
	Mean (mm)	SD	Range	Mean (mm)	SD	Range	
*BL*							
Labial	2.88	0.73	1.2–4.7	2.69	0.66	1.1–4.2	0.353
Palatal	0.97	0.43	0.3–1.9	0.75	0.36	0.1–1.6	0.055
*RL*							
Pretreatment	23.85	1.07	21.5–26.4	23.81	1.03	21.7–26.4	0.902
Posttreatment	22.29	1.12	20.1–24.5	22.34	1.16	20.3–25.3	0.89
ARR	1.56	0.69	0.4–3.0	1.48	0.57	0.5–2.7	0.648

*BL* bone loss, *RL* root length, *ARR* apical root resorption

In terms of the alveolar bone levels, the labial variations were far greater than those seen in the palatal bony plates in both the control and experimental groups (experimental group: labial BL = 2.88 ± 0.73 mm, palatal BL = 0.97 ± 0.43 mm; control group: labial BL = 2.69 ± 0.66 mm, palatal BL = 0.75 ± 0.36 mm). However, there were no statistically significant differences with regards to the alveolar bone levels between the control and experimental groups. The presence of ARR was common during the orthodontic treatment with the fixed appliances. The ARR ranged from 0.4 to 3.0 mm and was not statistically significant between the two groups (experimental group: 1.56 ± 0.69 mm, control group: 1.48 ± 0.57 mm; *P* = 0.648).

## Discussion

Tooth impaction was evaluated by clinical examination after confirmation of the absence of a permanent tooth in the dental arch within the expected time. CBCT revealed that the impacted tooth was located labial to the alveolar crest and provided information including: the proximity of the main anatomical structures, the accurate location and spatial structure of the impacted tooth and the presence of any obstacles. Transplantation is one solution to tooth impaction, however, an adequate volume of alveolar bone is essential and the long-term success rate of transplantation needs to be verified in a further study. Immediate implant placement after extraction is another alternative, although this has the disadvantage of additional surgery [23]. However, adolescents have to use temporary restorations before the implant insertion that risks potential problems [24].

Surgical exposure creates an eruption pathway for the impacted teeth and reduces the risk of some frequent complications related to untreated impacted teeth including: a dental cyst of the impacted tooth, ankylosis, malocclusion, root resorption of adjacent teeth and marginal bone loss of adjacent teeth [25]. Although surgical exposure is vital for impacted teeth to perform normal function, associated trauma is inevitable. Immediate damage might cause pain, inflammation and swelling, and the open wound is more vulnerable to the bacteria-bearing oral environment. In our study, patients reported no complications related to the surgical operation and there was no need for retreatment. The removal of bone tissue covering the crown is minimally invasive and preserves intrinsic tissue as much as possible. Resected mucosa, submucosa and muscle related to adhesion and scarring produce an effect on labial gingival aesthetics after tooth eruption. In our research, stitched DF and mucosa create a structural coverage that resembles a pocket to cover the wound. The remarkable effect of hemostasis reduces incisional drainage, and as a result the bonding of brackets is easier for the orthodontist. Since the epithelia of DF is continuous with the mucosa, tissue integrity is guaranteed in periodontium regeneration in tooth eruption.

An altered periodontium remolding pattern might give rise to PD and recession of the gingival margin, resulting in poor periodontal status. Nowzari et al*.* [6] reported that the width of keratinized tissue ranged from 2 to 6 mm (average 3.7 mm) in canines after orthodontic treatment. Our clinical observations present better outcomes of WKG. The PD of the impacted canines were statistically deeper than those of the contralateral canines only at the mesiolabial, distolabial and midpalatal sites, however, all outcomes were less than 3 mm.

Alveolar bone loss and apical root resorption occurs mainly in the anterior teeth in orthodontic treatment. A previous study reported that the surrounding bone decreases in the vertical direction on both the labial side (0.97 mm) and the lingual side (0.86 mm) in the movement of anterior teeth [26]. In our study, the labial variations were far greater than those in previous research (experimental group: 2.88 ± 0.73 mm; control group: 2.69 ± 0.66 mm). The difference in findings between this and the previous study is mainly due to the different methodologies used, including measurement methods, racial differences, and the measurement time. Postoperative follow-up time is too short to observe osteogenic activities. However, the differences showed no statistical significance between groups at a certain time when the fixed appliance was removed.

Teeth in different tooth positions present diverse susceptibility to persistent orthodontic force, and the maxillary incisors are considered to be the most affected. In this study, the maxillary canines are selected candidates on account of the second highest impaction rate with prevalence ranging from 0.27 to 2.4% among different ethnic groups [27]. Li et al. [28] investigated the ARR in erupted canine teeth treated with traditional fixed appliances and reported the prevalence and severity of ARR (80% and 1.53 ± 1.92 mm). In our research, the impacted canines did not develop significant ARR compared with the contralateral canines, therefore, our improved surgical method can be applied to impacted teeth in the anterior maxilla including incisors and lateral incisors.

The DF is considered essential for periodontal tissue repair and alveolar bone regeneration, therefore, surgeons should preserve the DF to the utmost extent in surgical exposure operations. Previous techniques take the crown-exposed area into account with the removal of partial or complete DF [6, 29]. During orthodontic traction, the open DF establishes a gateway to facilitate effective drainage of fluid when there is infection. Sufficient exposure of the crown achieves good visualization for orthodontists in the process of traction, making tooth eruption optimal and exerting controllable force.

Our team have performed 24 cases of labially impacted anterior maxillary canines with the surgical exposure technique described above. The collection of periodontal and bony measurements post-treatment is in progress due to the long follow-up time for orthodontic treatment. We would like to introduce this improvement in soft tissue management to elaborate the importance of DF for treatment efforts. Orthodontists and surgeons are equally important to treatment. A deeper communication and mutual participation between the two groups, both before initiating treatment and post-treatment, is needed to offset the cognitive bias from specialized knowledge.

To the best of our knowledge, no previously published study has investigated whether to reserve intact dental follicle in surgical exposure techniques in the treatment of impacted tooth and compared the periodontal remodeling. This study introduces the soft tissue management during surgical exposure of labially impacted maxillary canines and provides a quantitative analysis of the effects on aesthetics and periodontal health. Our technical improvement retains nearly all soft tissues with a minimum of bony tissue removal. In particular, the management of soft tissue is considered with the intention of achieving better aesthetic results, and post-treatment periodontal health. DF stitching to muscle and oral mucosa makes our technique minimally invasive, and with less exposed wound. The exposed crown simplifies the process of traction by achieving a visual pathway. Reserved DF plays an important role in the remolding of the alveolar bone with polarized performance of promoting bone resorption coronally, and accelerating bone formation apically [30]. The risk of inflammation and the degree of swelling post-surgery are minimized along with less pain. While this technology sets higher demands on surgeon’s abilities, accurate radiographic data makes postoperative assessment feasible. Within the limitations of this pilot study, our technique provided improved periodontal health and aesthetic outcomes.

## Conclusions

The preservation of DF promotes soft tissue management in combined surgical and orthodontic treatment of labially impacted maxillary canine to achieve better periodontal status.

## Data Availability

The data used in this study are available from the corresponding author on reasonable request.

## Abbreviations

CET: Closed-eruption technique
OET: Open-eruption technique
DF: Dental follicle
GI: Gingival index
PD: Probing depth
WKG: The width of the keratinized gingiva
GS: Gingival scars
BL: Bone loss
ARR: Apical root resorption
CBCT: Cone-beam computed tomography
CEJ: The cementoenamel junction
RANK: The receptor activator of nuclear factor kB
CSF-1: Colony-stimulating factor-1

## References

[1] Li R, Mei L, Wang P, He J, Meng Q, Zhong L, Zheng W, Li Y (2019). Canine edge width and height affect dental esthetics in maxillary canine substitution treatment. Prog Orthod.

[2] He D, Mei L, Wang Y, Li J, Li H (2017). Association between maxillary anterior supernumerary teeth and impacted incisors in mixed dentition. J Am Dent Assoc.

[3] Björksved M, Arnrup K, Lindsten R, Magnusson A, Sundell AL, Gustafsson A, Bazargani F (2018). Closed vs open surgical exposure of palatally displaced canines: surgery time, postoperative complications, and patients' perceptions: a multicentre, randomized, controlled trial. Eur J Orthod.

[4] Jiang Q, Yang R, Mei L, Ma Q, Wu T, Li H (2019). A novel approach of torque control for maxillary displaced incisors. Am J Orthod Dentofacial Orthop..

[5] Becker A, Chaushu S (2015). Surgical treatment of impacted canines: what the orthodontist would like the surgeon to know. Oral Maxillofac Surg North Am.

[6] Nowzari H, Rodriguez AE (2019). Impacted teeth: closed flap surgery. J Esthet Restor Dent.

[7] Mummolo S, Nota A, De Felice ME, Marcattili D, Tecco S, Marzo G (2018). Periodontal status of buccally and palatally impacted maxillary canines after surgical-orthodontic treatment with open technique. J Oral Sci.

[8] Chiara C, Papageorgiou SN, Theodore E (2018). Open versus closed surgical exposure for permanent impacted canines: a systematic review and meta-analyses. Eur J Orthodont..

[9] Vermette ME, Kokich VG, Kennedy DB (1995). Uncovering labially impacted teeth: apically positioned flap and closed-eruption techniques. Angle Orthod.

[10] Boyd RL (1984). Clinical assessment of injuries in orthodontic movement of impacted teeth. II. Surgical recommendations. Am J Orthod.

[11] Woloshyn H, Artun J, Kennedy DB, Joondeph DR (1994). Pulpal and periodontal reactions to orthodontic alignment of palatally impacted canines. Angle Orthod.

[12] Ericson S, Bjerklin K (2001). The dental follicle in normally and ectopically erupting maxillary canines: a computed tomography study. Angle Orthod.

[13] Zhou T, Pan J, Wu P, Huang R, Du W, Zhou Y, Wan M, Fan Y, Xu X, Zhou X, Zheng L, Zhou X (2019). Dental follicle cells: roles in development and beyond. Stem Cells Int.

[14] Sowmya S, Chennazhi KP, Arzate H, Jayachandran P, Nair SV, Jayakumar R (2015). Periodontal specific differentiation of dental follicle stem cells into osteoblast, fibroblast, and cementoblast. Tissue Eng Part C Methods.

[15] Dorotheou D, Gkantidis N, Karamolegkou M, Kalyvas D, Kiliaridis S, Kitraki E (2012). Tooth eruption: altered gene expression in the dental follicle of patients with cleidocranial dysplasia. Orthod Craniofac Res.

[16] Takahashi A, Nagata M, Gupta A, Matsushita Y, Yamaguchi T, Mizuhashi K, Maki K, Ruellas AC, Cevidanes LS, Kronenberg HM, Ono N, Ono W (2019). Autocrine regulation of mesenchymal progenitor cell fates orchestrates tooth eruption. P Natl Acad Sci.

[17] Yang B, Chen G, Li J, Zou Q, Xie D, Chen Y, Wang H, Zheng X, Long J, Tang W, Guo W, Tian W (2012). Tooth root regeneration using dental follicle cell sheets in combination with a dentin matrix—based scaffold. Biomaterials.

[18] Giannoudis PV, Dinopoulos H, Tsiridis E (2005). Bone substitutes: an update. Injury.

[19] Tavelli L, Barootchi S, Avila-Ortiz G, Urban IA, Giannobile WV, Wang HL (2021). Peri-implant soft tissue phenotype modification and its impact on peri-implant health: a systematic review and network meta-analysis. J Periodontol.

[20] Incerti-Parenti S, Checchi V, Ippolito DR, Gracco A, Alessandri-Bonetti G (2016). Periodontal status after surgical-orthodontic treatment of labially impacted canines with different surgical techniques: a systematic review. Am J Orthod Dentofacial Orthop.

[21] Gay G, Ravera S, Castroflorio T (2017). Root resorption during orthodontic treatment with Invisalign®: a radiometric study. Prog Orthod.

[22] Demmer RT, Jacobs DR, Desvarieux M (2008). Periodontal disease and incident type 2 diabetes: results from the first national health and nutrition examination survey and its epidemiologic follow-up study. Diabetes Care.

[23] García B, Boronat A, Larrazabal C, Peñarrocha M, Peñarrocha M (2009). Immediate implants after the removal of maxillary impactedcanines: a clinical series of nine patients. Int J Oral Maxillofac Implants.

[24] Jamilian A, Perillo L, Rosa M (2015). Missing upper incisors: a retrospective study of orthodontic space closure versus implant. Prog Orthod.

[25] Hermann L, Wenzel A, Schropp L, Matzen LH (2019). Impact of CBCT on treatment decision related to surgical removal of impacted maxillary third molars: does CBCT change the surgical approach?. Dentomaxillofac Rad.

[26] Guo R, Zhang L, Hu M, Huang Y, Li W (2020). Alveolar bone changes in maxillary and mandibular anterior teeth during orthodontic treatment: a systematic review and meta-analysis. Orthod Craniofac Res.

[27] Becker A, Chaushu S (2015). Etiology of maxillary canine impaction: a review. Am J Orthod Dentofacial Orthop.

[28] Li Y, Deng S, Mei L, Li Z, Zhang X, Yang C, Li Y (2020). Prevalence and severity of apical root resorption during orthodontic treatment with clear aligners and fixed appliances: a cone beam computed tomography study. Prog Orthod.

[29] Chapokas AR, Almas K, Schincaglia GP (2012). The impacted maxillary canine: a proposed classification for surgical exposure. Oral Surg Oral Med O.

[30] Marks SC, Cahill DR (1987). Regional control by the dental follicle of alterations in Alveolar bone metabolism during tooth eruption. J Oral Pathol.

